# Effects of dextromethorphan and oxycodone on treatment of neuropathic pain in mice

**DOI:** 10.1186/s12929-015-0186-3

**Published:** 2015-09-22

**Authors:** Pao-Pao Yang, Geng-Chang Yeh, Eagle Yi-Kung Huang, Ping-Yee Law, Horace H. Loh, Pao-Luh Tao

**Affiliations:** Graduate Institute of Medical Sciences, College of Medicine, Taipei Medical University, 250 Wuxing Street,, Taipei City, 110 Taiwan; Department of Pharmacology, National Defense Medical Center, 161, Sec. 6, Minquan E. Rd., Neihu Dist., Taipei City, 114 Taiwan; Department of Pharmacology, University of Minnesota, 6-120 Jackson Hall, 321 Church St. SE, Minneapolis, MN 55455-0217 USA; Center for Neuropsychiatric Research, National Health Research Institutes, 35 Keyan Road, Zhunan, Miaoli County 35053 Taiwan, ROC

**Keywords:** Oxycodone, Dextromethorphan, Spinal nerve ligation, Neuropathic pain, Allodynia

## Abstract

**Background:**

Neuropathic pain is a very troublesome and difficult pain to treat. Although opioids are the best analgesics for cancer and surgical pain in clinic, only oxycodone among opioids shows better efficacy to alleviate neuropathic pain. However, many side effects associated with the use of oxycodone render the continued use of it in neuropathic pain treatment undesirable. Hence, we explored whether dextromethorphan (DM, a known N-methyl-D-aspartate receptor antagonist with neuroprotective properties) could potentiate the anti-allodynic effect of oxycodone and underlying mechanisms regarding to glial cells (astrocytes and microglia) activation and proinflammatory cytokines release in a spinal nerve injury (SNL) mice model.

**Results:**

Oxycodone produced a dose-dependent anti-allodynic effect. Co-administration of DM at a dose of 10 mg/kg (i.p.) (DM10) which had no anti-allodynic effect by itself enhanced the acute oxycodone (1 mg/kg, s.c.) effect. When the chronic anti-allodynic effects were examined, co-administration of DM10 also significantly enhanced the oxycodone effect at 3 mg/kg. Furthermore, oxycodone decreased SNL-induced activation of glial cells (astrocytes and microglia) and plasma levels of proinflammatory cytokines (IL-6, IL-1β and TNF-α). Co-administration of DM10 potentiated these effects of oxycodone.

**Conclusion:**

The combined use of DM with oxycodone may have therapeutic potential for decreasing the effective dose of oxycodone on the treatment of neuropathic pain. Attenuation of the glial activation and proinflammatory cytokines in the spinal cord may be important mechanisms for these effects of DM.

## Background

Neuropathic pain is caused by a primary lesion or dysfunction in the somatosensory system. Symptoms of neuropathic pain may include hyperalgesia (increased sensitivity to noxious stimulus) and allodynia (in which low-threshold stimuli, such as brushing of the skin, can evoke pain) [1]. Neuropathic pain can be very difficult to treat with only 40–60 % of people achieving partial relief [2]. Some animal studies have suggested that activated microglia in spinal cord may play a vital role in nerve injury induced neuropathic pain [3, 4].

Opioid receptor agonists, such as morphine and oxycodone are highly effective strong analgesics for relief of moderate or severe pain. However, morphine is less effective in treating neuropathic pain. Chronic morphine exposure increased glial expression and enhanced proinflammatory cytokines in the L5 spinal cord of L5 nerve-injured rats. This enhanced glial expression followed by the loss of the analgesic effect of morphine (so called tolerance) [5]. Oxycodone is a semisynthetic opioid analgesic derived from a naturally occurring alkaloid, thebaine. It has been used in clinic since 1917 and is increasingly used worldwide to treat acute and chronic pain. Several reports have shown that oxycodone effectively relieved neuropathic pain in clinic [6, 7]. It is more effective than morphine in the mice models of painful diabetic neuropathy [7, 8] and sciatic nerve ligation-induced neuropathic pain [9]. Spinal nerve ligation induced down regulation of GABA_B_ expression which was prevented by inhibition of microglia activation in the spinal cord dorsal horn [10]. Recent study by Thibault et al. provided evidence that the long-term analgesic effect of oxycodone but not morphine is due to an up-regulation in GABA_B_ receptor expression in sensory neurons and subsequently reinforce a presynaptic inhibition [11]. Therefore, the first aim of our study was to investigate whether chronic oxycodone treatment could suppress the glial activation and proinflammatory cytokines in a mice model of spinal nerve injury.

Dextromethorphan (DM) has been used in clinics as an antitussive agent (15–30 mg, 3 to 4 times per day in adult) for more than 50 years [12]. It can act as low affinity non-competitive N-methyl-D-aspartate (NMDA) receptor antagonist [13], α3β4-nicotinic receptor antagonist [14], and sigma-1 receptor agonist [15]. Our previous studies showed that DM could effectively reduce the rewarding effects (i.e., addiction potential) and drug-seeking effects of morphine [16, 17] or methamphetamine [18, 19] in rats. DM also has important neuroprotective properties in various CNS injury models, including ischemia, seizure, and traumatic brain injury paradigms [20]. Co-administration of L-NAME (nitric oxide synthase inhibitor) and DM prevented pathological pain in sciatic nerve ligation induced neuropathy in the chronic constriction injury (CCI) model [21]. DM blocks LPS-induced microglial activation in a concentration-dependent manner in vitro [22, 23] and also inhibits methamphetamine-induced microglial activation in vivo [22]. Many of these effects of DM seem functionally related to its inhibitory effects on glutamate-induced neurotoxicity via NMDA receptors or voltage-gated calcium channel activities [24]. Since oxycodone still has high abused and addictive potential and all the side effects of opiates at the therapeutic doses, the second aim of our study was to investigate whether DM at a dose that did not have anti-allodynic effect by itself could potentiate the effect of oxycodone on treatment of neuropathic pain and therefore decrease the effective dose of oxycodone in a mice model. The underlying mechanisms regarding to suppression of glia activation and proinflammatory cytokines were also investigated in the present study.

## Methods

### Animals

One hundred and twenty three adult male C57BL/6 J mice (25–30 g; from mating of the parental C57BL/6 J strain) were used in this study. The animals were kept in an animal room with a 12-h light/dark cycle, at a temperature of 25 ± 2 °C and humidity of 55 % at the Animal Center of Taiwan’s National Defense Medical Center, which is accredited by AAALAC International. Standard diet and water were provided ad libitum during the experiment. The care of animals was carried out in accordance with institutional and international standards (Principles of Laboratory Animal Care, NIH), and the protocol was approved by the Institutional Animal Care and Use Committee (IACUC) of National Defense Medical Center, Taiwan, ROC. All studies involving animals are reported in accordance with the ARRIVE guidelines [25].

### Spinal nerve ligation (SNL) surgery

L5 SNL was carried out according to a previously described method for rats [26] and modified for mice in our laboratory [27]. The mice were deeply anesthetized with sodium pentobarbital (80 mg/kg, i.p.), and the hairs on their back were clipped. A midline incision above the lumbar spine exposed the left sixth lumbar transverse process. The transverse process was removed carefully with a small scraper. The underlying fifth lumbar nerve root was isolated and then tightly ligated with 8–0 nylon thread. Next, the wound was closed with 2–3 muscle sutures (3–0 absorbable nylon suture) and 4–5 skin sutures (3–0 non-absorbable nylon suture). The surgical procedure for the sham group was identical, except that the fifth lumbar spinal nerve was not ligated and transected.

### Experimental schedule and groups

**Fig. 1:**
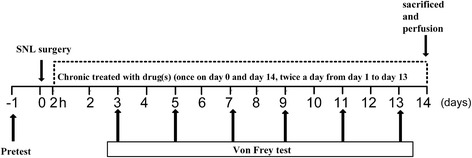
The schedule of the experiments

As shown in Fig. 1, a 14-day schedule was used in this study. Drug(s) was/were administered from 2 h after spinal nerve ligation surgery in mice (once on day 0 and day 14, twice a day from day 1 to day 13). Von Frey tests were used for measuring mechanical allodynia every other day. Two tests were carried out in mice on each test day. The first one was tested 1 h before drug(s) administration, in order to see the development of allodynia under chronic drug(s) treatment. The second test was tested 30 min after drug(s) administration in order to see the acute anti-allodynic effect of drug(s). There were more than 10 groups based on different drug(s) treatment, such as sham operation with saline treatment (sham + saline), SNL surgery with saline treatment (SNL + saline), SNL surgery with DM treatment (10 mg/kg, i.p. or 20 mg/kg, i.p.) (SNL + DM10, SNL + DM20), SNL surgery with oxycodone treatment (1, 3, or 5 mg/kg, s.c.) (SNL + O1, SNL + O3 or SNL + O5), and SNL surgery with co-treatment with oxycodone (1 or 3 mg/kg, s.c.) and DM (10 mg/kg, i.p.) (SNL + O1 + DM or SNL + O3 + DM). The animals were given the last drug administration 30 min before sacrificed on day 14 for immunohistochemistry study. The number of animals in each group was at least 8 at the beginning of the experiment; and at least 5 mice in each group survived to complete this study.

### Von Frey test for determination of mechanical allodynia

The mice were individually placed in a transparent acrylic box (9 × 9 × 15 cm) with a wire-mesh bottom and allowed to acclimate to their environment for at least 30 min. The mechanical stimulus was applied from underneath to the plantar aspect of the hind limb, with a gradual increase in pressure by means of an Electronic von Frey apparatus (IITC Inc., CA, USA). The end point was characterized by the removal of the paw followed by clear flinching movements. After the paw withdrawal, the intensity of the pressure was automatically recorded. The test was carried out 1 h before (pretest) and 30 min after saline or drug(s) injection. Each test was repeated 3 times with intervals of 5 min, and the average value was used. The area under the time-effect curve (AUC) was calculated for each animal according to the following formula: |(paw withdrawal pressure of each time point) – (paw withdrawal pressure of pre-operative baseline value)| × time (days).

### Immunohistofluoresensce for activated spinal astrocytes or microglia

Mice were anesthetized with pentobarbital (80 mg/kg i.p.) 30 min after saline or drug administration on day 14 and perfused with Tyrode’s calcium-free buffer (116 mM NaCl/5.36 mM KCl/1.57 mM MgCl_2_.6H_2_O/0.405 mM MgSO_4_/1.23 mM NaH_2_PO_4_/5.55 mMglucose/26.2 mM NaHCO_3_, pH 7.4), followed by 4 % paraformaldehyde in 0.1 M phosphate buffer (pH 7.4). The L4-L6 spinal segments were carefully removed, post-fixed in the same fixative for 2 h at 4 °C, and then placed in 30 % sucrose solution for 48–72 h at 4 °C. The samples were then embedded in OCT compound and frozen immediately at −80 °C. Serial transverse spinal cord slices (10 μm) were sectioned with a cryostat. The slices were mounted on SuperFrost Plus slides (Menzel-Glaser) and were air-dried for 30 min at room temperature, and washed three times in ice-cold phosphate-buffered saline Tween-20 (PBST). The slices were then fixed by immersing the slides in acetone/methanol (1:1, pre-cooled to −20 °C) for 3 min and washing them again three times in ice-cold PBST. The sections were then pre-incubated with blocking buffer (3 % normal goat serum diluted in PBST) for 1 h at room temperature. After washing in ice-cold PBST, the sections were incubated overnight at 4 °C with mouse monoclonal anti-GFAP antibody (1:400; Millipore, Cat #MAB360) or incubated over 2 nights with rabbit anti-iba1 antibody (1:300; Wako), which was diluted in PBS. Following primary antibody incubation, the sections were washed three times with PBST and incubated with the secondary antibody [goat anti-mouse FITC conjugated (1:100, Millipore, Cat #AP124F), or Alexa Fluro 488 goat anti-rabbit IgG (1:200), which were diluted with blocking buffer] for 1 h at room temperature. Sections were then washed with PBST, cleared, and cover-slipped by using mounting medium (Serotec, HIS002B). The processed sections were captured using a Leica (DMI 6000B) inverted microscope and a Leica (DFC 420) camera by MetaMorph software (Major Instruments Co., Ltd). Five spinal cord sections from the L5 segments were randomly selected from each mice. So in each group, there were 30 sections obtained for histology quantifications. Images were evaluated by a computer-assisted image analysis program (MetaMorph 6.1). Our image data were collected using the same region and the same size of field within same lamina to avoid any variance and difference in staining between lamina. The immunoreactivities for GFAP and iba1 immunopositive cells within the superficial dorsal horn were averaged the spinal sections for each experimental group.

### Quantification of cytokine levels using xMAP technology

Blood samples were collected on day 2 and day 6 after spinal nerve ligation surgery in mice. Blood was mixed with 1.8 mg/ml EDTA and put on ice for less than 10 min. Plasma was then collected by centrifugation at 1000 g for 10 min at 4 °C and stored at −80 °C until analysis. Cytokine levels were determined using the Milliplex MAP mouse cytokine/chemokine kit (Millipore, Billerica, MA, USA; MCYTOMAG-70 K), with specific bead sets – pro-inflammatory cytokines (IL-6, IL-1β, and TNF-α). The data acquisition and analysis were performed using Milliplex Analyst 5.1.

### Statistical analysis

The data were expressed as means ± S.E.M. Student’s t test, two-way or one-way ANOVA, Bonferroni post hoc test, or Newman-Keuls test were used to analyze the data. A difference was considered to be significant at *p* < 0.05.

## Results

### Effects of oxycodone on SNL-induced allodynia

**Fig. 2:**
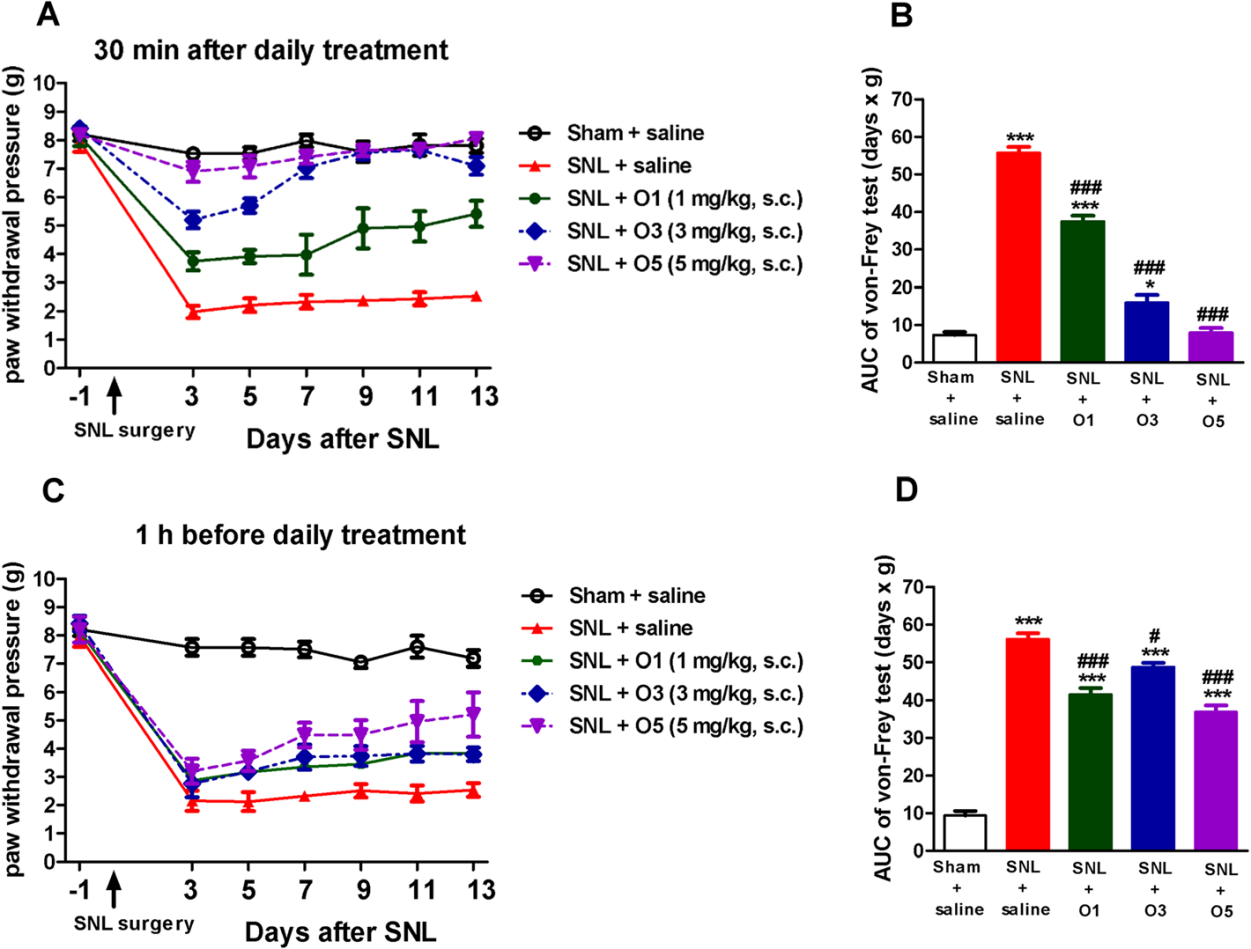
Oxycodone produced a significant and dose-dependent acute effect on SNL-induced allodynia. The ipsilateral paw withdrawal pressure was determined every other day after SNL by von Frey test. (**a**) acute effect determined 30 min after daily saline or oxycodone administration, or (**c**) chronic effect determined 1 h before saline or oxycodone administration. The area under curve (AUC) values of corresponding curves of the von Frey tests are shown in (**b**) and (**d**). Data are presented as means ± S.E.M. (*n* = 5–9). One-way ANOVA and Newman-Keuls test were used to analyze the data. ^*^ *P* < 0.05, ^***^ *p* < 0.001 vs. Sham + saline group; ^#^ *p* < 0.05, ^###^ *p* < 0.001 vs. SNL + saline group

We first explored the effects of oxycodone on the mechanical allodynia induced by SNL. The withdrawal pressure for ipsilateral hind paw was significantly decreased from 7–9 g to around 2 g on day 3 after SNL surgery (SNL + saline group in Fig. 2a). On the other hand, the sham operation did not alter the ipsilateral hind paw withdrawal pressure at all (sham + saline group in Fig. 2a). These results indicate that the allodynia of the ipsilateral hind paw was induced by the ligation of the L5 spinal nerve. The withdrawal pressure of the contralateral side of the nerve-ligated group did not change significantly through the time course (data not shown).

Furthermore, the withdrawal pressure of the ipsilateral hind paw was determined 1 h before and also 30 min after saline or oxycodone treatment (1, 3, or 5 mg/kg, s.c.) every other day. There was a dose-dependent acute anti-allodynic effect of oxycodone when tested 30 min after drug administration, as shown in Fig. 2a. Oxycodone at a dose of 5 mg/kg (s.c.) completely blocked the allodynia of the ipsilateral hind paw in all test days. The corresponding area under curve (AUC) of the time-effect curves also showed that the oxycodone had a dose-dependent anti-allodynia effect (Fig. 2b). However, this acute effect of oxycodone did not persist. When we measured the withdrawal pressure of the ipsilateral hind paw 1 h before drug treatment on test days, though there was significant attenuation of the allodynia, the chronic effects of oxycodone were much less than the acute effects of oxycodone [Fig. 2c, d vs. Fig. 2a, b]. These data indicate that oxycodone (1, 3, or 5 mg/kg, s.c.) not only had an acute anti-allodynic effect but also could improve the chronic course of the SNL-induced allodynia.

### Effects of DM on SNL-induced mechanical allodynia

**Fig. 3:**
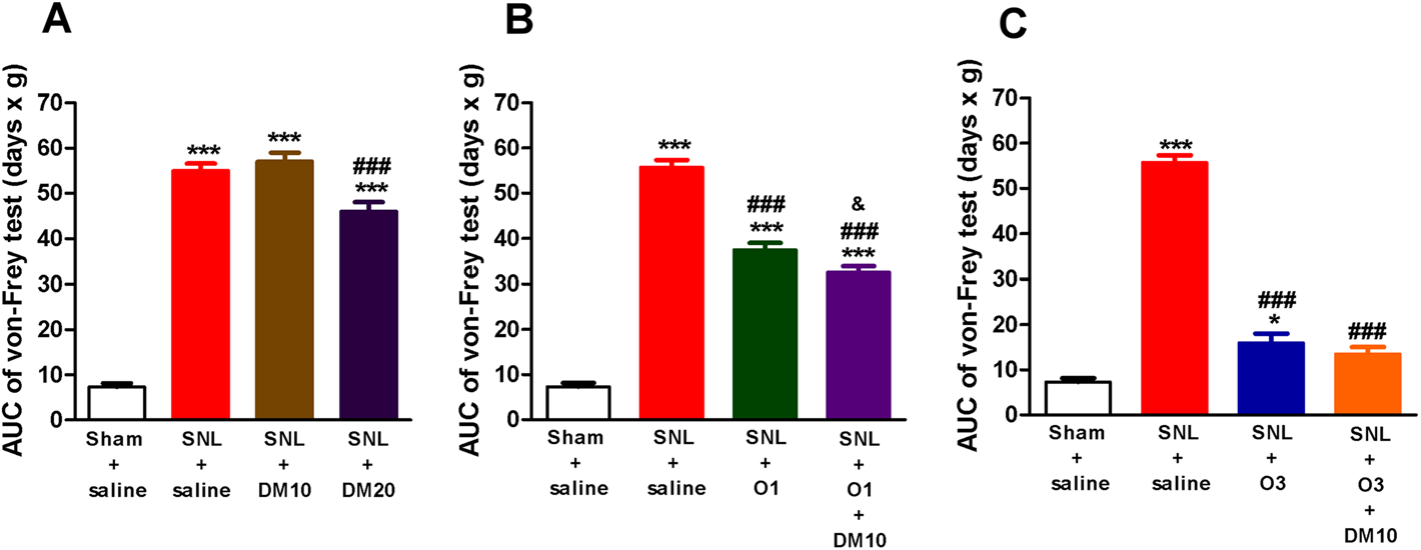
DM (10 mg/kg, i.p.) enhanced the acute effect of oxycodone (1 mg/kg, s.c.) on SNL-induced allodynia. The ipsilateral paw withdrawal pressure was determined 30 min after saline or drug administration every other day after SNL. (**a**) effects of DM10 (DM, 10 mg/kg, i.p.) or DM20 (DM, 20 mg/kg, i.p.); (**b**) effects of O1 (oxycodone, 1 mg/kg, s.c.) or O1 + DM10; (**c**) effects of O3 (oxycodone, 3 mg/kg, s.c.) or O3 + DM10. Data are presented as means ± S.E.M. (*n* = 5–9). One-way ANOVA and Newman-Keuls test were used to analyze the data. ^*^ *P* < 0.05, ^***^ *p* < 0.001 vs. Sham + saline group; ^###^ *p* < 0.001 vs. SNL + saline group; ^&^ *p* < 0.05 vs. SNL + O1 group

**Fig. 4:**
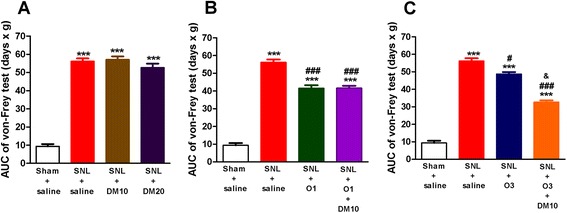
DM (10 mg/kg, i.p.) enhanced the chronic effects of oxycodone (3 mg/kg, s.c.) on SNL-induced allodynia. The ipsilateral paw withdrawal pressure was determined every other day after SNL at about 1 h before daily saline or drug administration. (**a**) effects of DM10 (DM, 10 mg/kg, i.p.) or DM20 (DM, 20 mg/kg, i.p.); (**b**) effects of O1 (oxycodone, 1 mg/kg, s.c.) or O1 + DM10; (**c**) effects of O3 (oxycodone, 3 mg/kg, s.c.) or O3 + DM10. Data are presented as means ± S.E.M. (*n* = 5–9). One-way ANOVA and Newman-Keuls test were used to analyze the data. ^***^ *P* < 0.001 vs. Sham + saline group; ^#^ *p* < 0.05, ^###^ *p* < 0.001 vs. SNL + saline group; ^&^ *p* < 0.05 vs. SNL + O3 group

We next investigated the effects of DM by itself on the mechanical allodynia induced by SNL. As shown in Fig. 3a, 20 mg/kg of DM (i.p.) produced a minimal but significant anti-allodynia effect in SNL mice (Fig. 3a, SNL + DM20 vs. SNL + saline group; *p* < 0.001). On the other hand, 10 mg/kg of DM did not produce any acute anti-allodynia effect after daily drug treatment in SNL mice (SNL + DM10 vs. SNL + saline group). Neither 10 mg/kg nor 20 mg/kg of DM by itself showed significant chronic effects when tests were done 1 h before drug treatment, as shown in Fig. 4a. Hence, in the following studies, we chose a dose of DM that by itself had no acute anti-allodynic effect, i.e., 10 mg/kg (i.p.), to co-administer with different submaximal doses of oxycodone (i.e., 1 or 3 mg/kg, s.c.) so as to investigate whether DM could enhance the anti-allodynic effect of oxycodone.

### Co-administration of DM with oxycodone on SNL-induced allodynia

The acute anti-allodynic effect of oxycodone was dose-dependent, as previously shown in Fig. 2b. When mice were treated with drug(s) after SNL (twice a day from day 1 to day 13) and the Von Frey tests were done every other day, we found that oxycodone by itself at a lower dose, i.e., 1 mg/kg, improved the allodynia partially, as shown in the Fig. 3b. Co-administration of DM (10 mg/kg, i.p.) enhanced the acute effect of oxycodone (1 mg/kg, s.c.) minimally but significantly (Fig. 3b, *p* < 0.05). However, this combined treatment did not improve the chronic effect of oxycodone (1 mg/kg, s.c.) if tested 1 h before oxycodone administration every other day, as shown in Fig. 4b.

The acute anti-allodynic effects of oxycodone at a higher dose of 3 mg/kg were shown in Fig. 3c. It could be seen that the AUC value of oxycodone (3 mg/kg) was much less than that in the SNL + saline group (*p* < 0.001) but still larger than that in the sham + saline group (Fig. 3c, *p* < 0.05). There is an apparent enhancement of anti-allodynic effects of 3 mg/kg oxycodone with the co-administration of DM (10 mg/kg) as demonstrated by no significant difference in the AUC value between this group and that of the sham + saline group (Fig. 3c). More importantly, when the Von Frey tests were done 1 h before drug treatment every other day, co-administration of DM (10 mg/kg) with 3 mg/kg of oxycodone significantly enhanced the chronic anti-allodynic effects of oxycodone, as shown in Fig. 4c, suggesting that this combined use of DM (10 mg/kg) with oxycodone (3 mg/kg, s.c.) could further alleviate the development of chronic allodynia.

### Oxycodone suppressed the SNL-induced activation of astrocytes and microglia

**Fig. 5:**
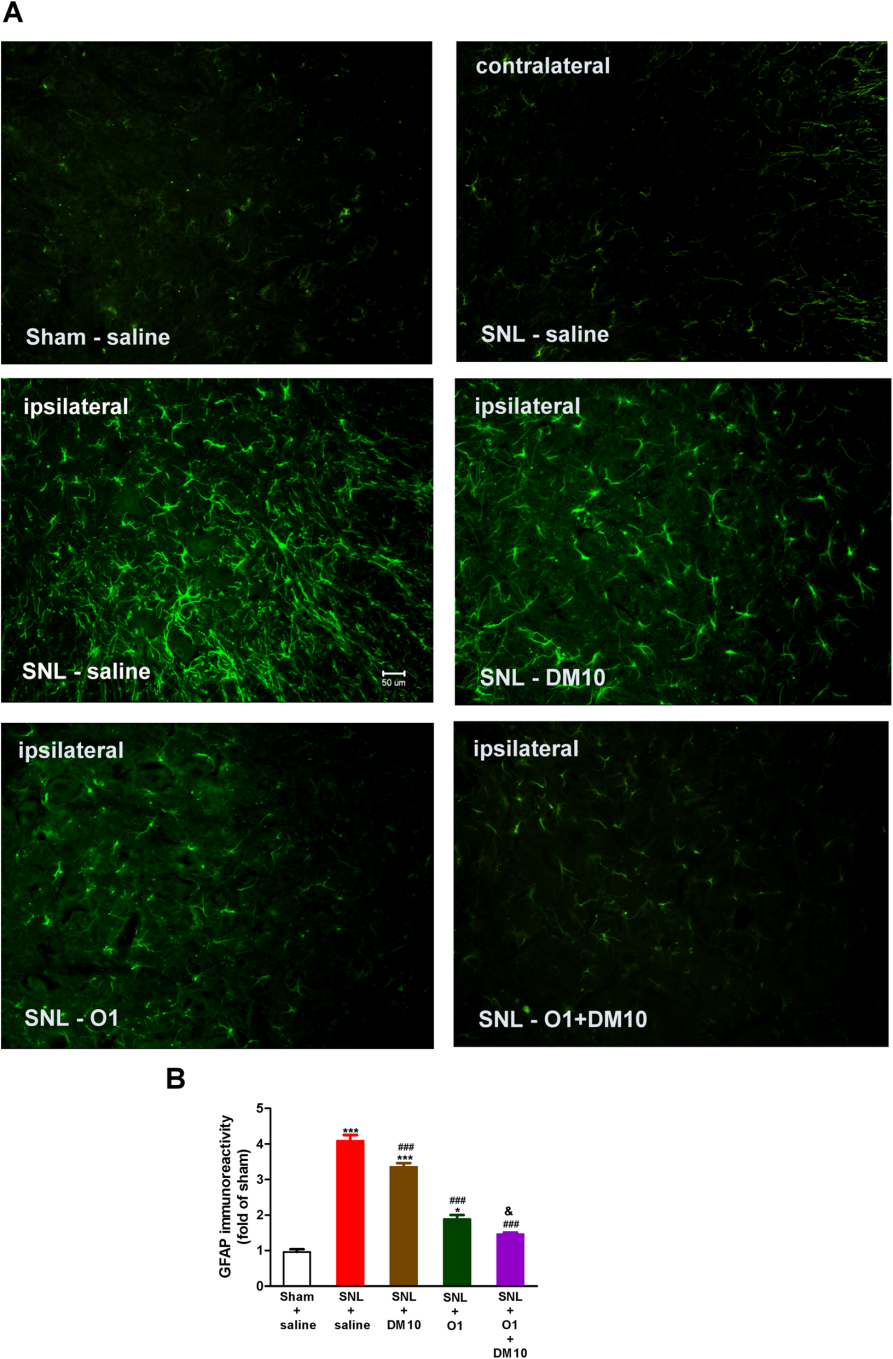
Co-administration of DM (10 mg/kg, i.p.) and oxycodone (1 mg/kg, s.c.) enhanced the effect of oxycodone to suppress SNL-induced activation of astrocytes in the L5 spinal cord dorsal horn. (**a**) Representative immunofluorescent images of astrocytes stained with GFAP (green; marker for activated astrocyte) on day 14 is shown for the following groups: Sham (ipsilateral), SNL (contralateral), SNL (ipsilateral), SNL-DM10 (DM 10 mg/kg, i.p.; ipsilateral), SNL-O1 (oxycodone 1 mg/kg, s.c.; ipsilateral), and SNL-O1 + DM10 (co-administration of oxycodone and DM; ipsilateral) (magnification: 20X). Scale bar, 50 μm. (**b**) Quantification of GFAP-immunoreactivity after normalization for each group compared with control group (Sham + saline) on day 14. Data are presented as means ± S.E.M. (n ≥ 5). One-way ANOVA and Newman-Keuls test were used to analyze the data. ^*^ *P* < 0.05, ^**^ *p* < 0.01, ^***^ *p* < 0.001 vs. Sham + saline group; ^#^ *p* < 0.05, ^###^ *p* < 0.001 vs. SNL + saline group; ^&^ *p* < 0.05 vs. SNL + O1 group

Morphological changes of astrocytes and microglia during allodynia under SNL-induced neuropathic pain were examined by carrying out immunohistofluoresence studies. In the sham-operated group (sham + saline), GFAP-immunopositive astrocytes had a less ramified morphology. In contrast, SNL induced significant activation of astrocytes manifested by a strong GFAP immunoreactivity and hypertrophic morphology in the dorsal spinal cord (L5) of ipsilateral side, which could be seen on day 14 after SNL surgery (Fig. 5a). Quantification of immunoreactivity by measuring the intensity of the staining indicated significant increase of GFAP immunoreactivity by SNL (4.1-fold on day 14), as compared with the sham control group (sham + saline) (Fig. 5b). Daily treatment with oxycodone (1 mg/kg) significantly suppressed SNL-induced activation of astrocytes (Fig. 5a, b).

**Fig. 6:**
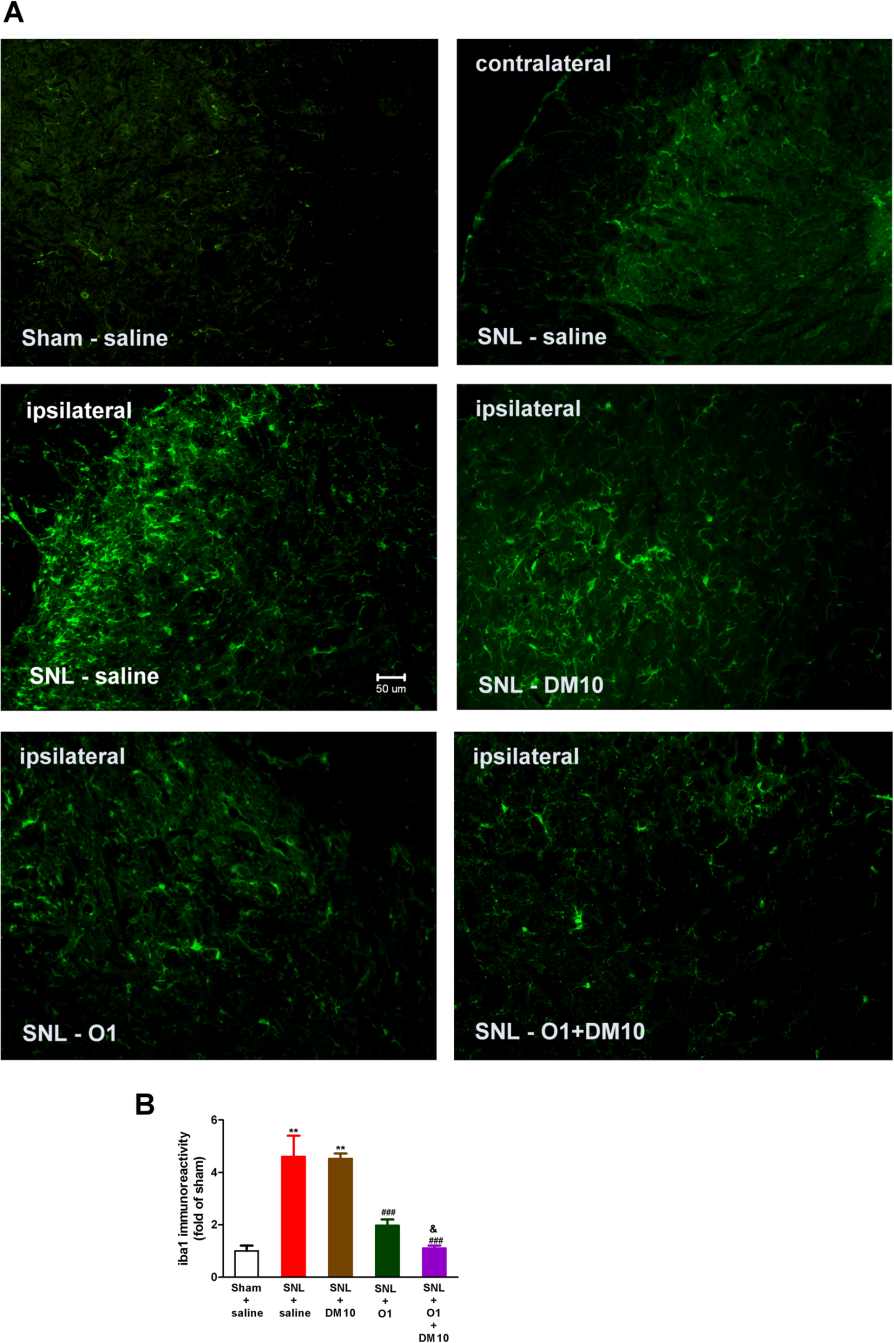
Co-administration of DM (10 mg/kg, i.p.) and oxycodone (1 mg/kg, s.c.) enhanced the effect of oxycodone to suppress SNL-induced activation of microglia in the L5 spinal cord dorsal horn. (**a**) Representative immunofluorescent images of microglia stained with iba1 (green; marker for activated microglia) on day 14 is shown for the following groups: Sham (ipsilateral), SNL (contralateral), SNL (ipsilateral), SNL-DM10 (DM 10 mg/kg, i.p.; ipsilateral), SNL-O1 (oxycodone 1 mg/kg, s.c.; ipsilateral), and SNL-O1 + DM10 (co-administration of oxycodone and DM; ipsilateral) (magnification: 20X). Scale bar, 50 μm. (**b**) Quantification of iba1-immunoreactivity after normalization for each group compared with control group (Sham + saline) on day 14. Data are presented as means ± S.E.M. (*n* ≥ 5). One-way ANOVA and Newman-Keuls test were used to analyze the data. ^*^ *P* < 0.05, ^**^ *p* < 0.01, ^***^ *p* < 0.001 vs. Sham + saline group; ^#^ *p* < 0.05, ^###^ *p* < 0.001 vs. SNL + saline group; ^&^ *p* < 0.05 vs. SNL + O1 group

The microglia with a resting ramified morphology by staining with a marker (iba1) were observed at the ipsilateral side of the dorsal spinal cord of sham-operated mice on day 14 (Fig. 6a). On the other hand, hypertrophic and amoeboid morphology of microglia in SNL mice were seen on day 14, indicating that microglia were more activated in SNL mice than those in sham-operated mice (Fig. 6a). The immunoreactivity of iba1 was also markedly increased in the ipsilateral L5 spinal cord dorsal horn (4.6-fold increase) after spinal nerve ligation for 14 days (SNL + saline group), as compared with the sham control group (sham + saline) (Fig. 6b). Oxycodone (1 mg/kg) significantly suppressed SNL-induced microglia activation, as shown in Fig. 6b.

### DM potentiated the effect of oxycodone in suppressing the SNL-induced activation of astrocytes and microglia

Daily treatment with DM (10 mg/kg) by itself slightly but significantly suppressed SNL-induced activation of astrocytes (Fig. 5a). After daily co-administration of oxycodone (1 mg/kg) with DM (10 mg/kg), some activated astrocytes were reversed to the resting state, as indicated by a further decrease in GFAP-positive cells (Fig. 5a, b).

DM (10 mg/kg) alone did not suppress the SNL-induced microglia activation, as shown in Fig. 6b. However, co-administration of DM with oxycodone potentiated the effect of oxycodone and further inhibited the SNL-induced microglia activation to the control (sham + saline) level (Fig. 6b). These results suggest that DM can enhance the effects of oxycodone (1 mg/kg) in suppressing the SNL-induced activation of both microglia and astrocytes. Since this effect of DM + O1 has reached the maximal effect, we did not further do the immunostaining for DM + O3 group.

### The plasma level of SNL-induced pro-inflammatory cytokines was reduced by oxycodone or co-administration of DM with oxycodone

**Fig. 7:**
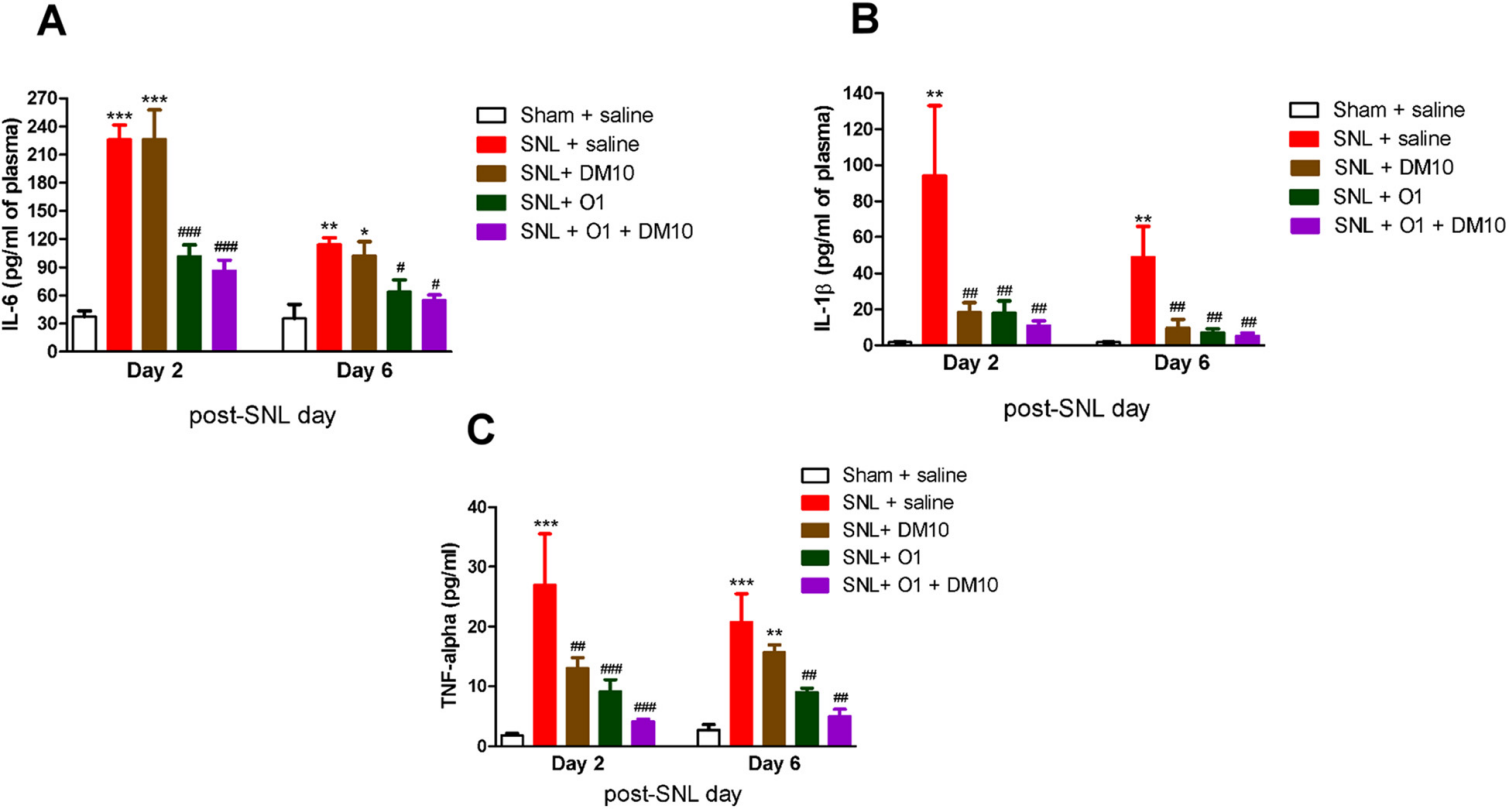
Effects of DM (10 mg/kg, i.p.) and oxycodone (1 mg/kg, s.c.) on SNL-induced plasma level of pro-inflammatory cytokines: (**a**) IL-6, (**b**) IL-1β, and (**c**) TNF-α on day 2 (D2) and day 6 (D6) after SNL surgery. Data are presented as means ± S.E.M. (*n* = 7–12). One-way ANOVA and Newman-Keuls test were used to analyze the data. ^*^ *P* < 0.05; ^**^ *p* < 0.01; ^***^ *p* < 0.001 vs. Sham + saline group; ^#^ *p* < 0.05; ^##^ *p* < 0.01; ^###^ *p* < 0.001 vs. SNL + saline group

Spinal-nerve injury induces rapid production and release of proinflammatory mediators [28, 29]. Therefore, whether co-administration of DM with oxycodone would enhance the effect of oxycodone on pro-inflammatory cytokines in plasma for each group on day 2 and day 6 after SNL surgery were determined. As shown in Fig. 7, SNL induced a significantly higher plasma level in all three pro-inflammatory cytokines we measured — i.e., interleukin-6 (IL-6, Fig. 7a), interleukin-1β (IL-1β, Fig. 7b), and tumor necrosis factor alpha (TNF-α, Fig. 7c) — on day 2 and day 6 after spinal nerve ligation. These increases were suppressed by either the treatment of oxycodone alone (1 mg/kg) or co-administration of DM (10 mg/kg) with oxycodone (1 mg/kg) (Fig. 7a, b, c). DM (10 mg/kg) by itself reduced the plasma concentration of IL-1β (Fig. 7b) and TNF-α (Fig. 7c) on day 2 and/or day 6 after surgery, but did not affect the plasma level of IL-6. When DM (10 mg/kg) was co-administered with oxycodone, it seemed to further decrease the plasma levels of IL-1β and TNF-α, which were already suppressed by oxycodone (1 mg/mg), although this did not achieve statistical significance.

## Discussion

The present study demonstrates that acute administration of oxycodone (1–5 mg/kg, s.c.) suppressed SNL-induced mechanical allodynia in a dose-dependent manner. DM at a low dose (10 mg/kg, i.p.) potentiated this acute effect of oxycodone. More importantly, it also alleviated the development of chronic allodynia better than use of oxycodone (medium dose, 3 mg/kg, s.c.) alone. We found that chronic oxycodone significantly suppressed the activation of astrocytes and microglia in the spinal cord and also the plasma level of proinflammatory cytokines (IL-6, IL-1β and TNF-α). Furthermore, co-administration of DM with oxycodone showed better effects than oxycodone alone in suppressing the SNL-induced activation of astrocytes and microglia in the dorsal horn of spinal cord and also showed a trend to enhance the effect of oxycodone on decreasing the plasma level of proinflammatory cytokines.

Oxycodone is an agonist acting on opioid receptors which are all coupled to the G proteins and distributed in the central and peripheral nervous system [30]. Oxycodone has been used in clinic for the treatment of cancer and neuropathic pain for many years [6]. Similar to other opiate drugs, oxycodone at high doses may have many side effects, including sedation, dizziness, nausea, vomiting, constipation, respiratory depression, dependence, and tolerance.

After peripheral nerve injury, spinal sensory neurons (DRG neurons) increased sodium-channel expression on sensitized primary afferents leads to increase of glutamate release from the nerve endings. Glutamate, released from pre-synaptic terminal, binds post-synaptic ionotropic and metabotropic receptors, leading to calcium influx through NMDA and AMPA receptors and activate protein kinase which may contribute to increase gain in the pain transmission system [31]. DM is a commonly used antitussive drug. It acts as a low-affinity non-competitive NMDA receptor antagonist and a high affinity sigma-1 receptor agonist, and it suppresses glutamate-induced excitotoxicity in the CNS and spinal regions [13]. Structurally, it is closely related to levorphanol, codeine, and morphine, but is dextrorotary in form. Therefore, it has low affinity for opioid receptors and is not considered to be addictive. NMDA receptor antagonists (such as MK-801 and ketamine) and sigma receptor agonists have been reported to have anti-inflammatory effects [32, 33]. In our results, acute administration of DM alone at a dose of 10 mg/kg (i.p.) did not show a significant anti-allodynic effect in SNL mice, whereas acute administration of DM at a dose of 20 mg/kg (i.p.) elicited a slight but significant anti-allodynic effect in this model (Fig. 3a). Recently, several clinical studies have shown that high doses of DM (270–400 mg/day) have analgesic effects to neuropathic pain with traumatic origin or diabetic neuropathy, but not with post-herpetic neuralgia [34, 35]. Additionally, low doses of DM (90 mg/day or less) did not relieve chronic neuropathic pain [36] or neuropathic pain resulting from cancer [37]. In rat studies, DM (40 mg/kg, i.p.) and its metabolite dextrophan (20–40 mg/kg, i.p.) induced PCP-like side effects (memory and psychotomimetic disturbances); but 10–30 mg/kg (i.p.) of DM did not impair reference memory [38]. Albers et al. (1992) reported that adverse effects of DM seem to be dose related [39]. It is well known that DM is metabolized by O-demethylation through cytochrome P450 2D6 (CYP2D6) to form its active metabolite, dextrophan, and by N-demethylation to form 3-methoxymorphinan, through CYP3A4 [40]. Dextrophan and 3-methoxymorphinan is then further N-demethylation and O-demethylation through CYP3A4 and CYP2D6 to form 3-hydroxymorphinan [41]. Therefore, use of high doses of DM may produce phencyclidine (PCP)-like effects from the metabolic conversion of DM to its metabolite, dextrophan. In the current study, we selected a low dose of DM (10 mg/kg, i.p.; DM10), which did not produce a significant anti-allodynic effect by itself, so that we could investigate whether it would potentiate the acute and/or chronic effects of oxycodone on suppressing neuropathic pain with minimal side effects.

The acute effects of drug(s) in this study were tested 30 min after drug(s) administration every other day. DM10 could potentiate the acute anti-allodynic effects of oxycodone (1 mg/kg) when tested 30 min after drug administration as shown in Fig. 3b. Since the acute anti-allodynic effects of oxycodone at higher dose (3 mg/kg) was already very good, we could not see further potentiated effect of DM10 (Fig. 3c) at this dose of oxycodone. Although we did not carry out the pharmacokinetic study in mice, we surmise that the acute effects would not last long because the half-life (t_1/2_) for the elimination of oxydocone is only 0.52 ± 0.04 h in S.D. rats [42] and 3.7 ± 2.3 h in humans [43]. Therefore, when we tested for the oxycodone effect about 1 h before daily drug(s) administration in the morning, this can serve as a measure for the long-term effects of drug(s) on SNL-induced neuropathic pain. In terms of treatment of chronic neuropathic pain, this is a more important measure than the acute effects of drug(s) and we found that combined treatment of DM10 with 3 mg/kg of oxycodone significantly alleviate chronic allodynia, better than oxycodone alone (3 mg/kg, s.c.) (Fig. 4c). We did not see the potentiated anti-allodynia effect of DM on 1 mg/kg of oxycodone (Fig. 4b) when we tested 1 h before drug administration. It certainly needs further investigation.

It has been reported that spinal damage and peripheral nerve damage both lead to physiological and morphological activation of glial cells, particularly astrocytes and microglia [44]. This activation could be initiated by the release of proinflammatory cytokines (IL-1β, IL-6 and TNFα), glutamate and nitric oxide (NO) from glial cells. These mediators can initiate and maintain neuropathic pain, inducing hyperexcitability of nociceptive neurons in the spinal cord dorsal horn. Increased expression of astrocyte markers (i.e., GFAP) and microglia markers (iba1) have been observed in many neuropathic pain models, such as chronic constriction injury (CCI), partial sciatic nerve ligation (PSNL) [45], and spared nerve injury (SNI) [46]. Ma & Quirion also reported a slight increase in GFAP immunoreactivity of astrocytes on the ipsilateral L4-5 spinal cord dorsal horn one week after PSNL, but a dramatic increase of GFAP immunoreactivity 3 weeks after PSNL [47]. The activation of glial was shown to be directly involved in neuropathic pain, since glial inhibitors exhibited anti-allodynic properties [48]. In our current study, both astrocytes and microglia activation induced by SNL caused changes in morphology, and increased the immunoreactivity of GFAP and iba1 at the ipsilateral spinal cord dorsal horn on day 14 after spinal nerve ligation (Figs. 5 and 6). We did not determine the activation of astrocytes or microglia during the early period after SNL, because the chronic effects of drug(s) could be seen only after 3 days of SNL. Chronic oxycodone treatment (1 mg/kg, s.c.) significantly decreased the activated astrocytes and microglia as we observed on day 14. A recent study has shown that oxycodone maintained a long-term and strong analgesic effect whereas the analgesic effect of morphine gradually decreased after chronic treatment. This long-term analgesic effect of oxycodone was due to up-regulation of GABA_B_ receptor expression in DRG neuron under vincristine-induced neuropathic pain in rats [11]. A recent report has shown that spinal nerve ligation induced significant decrease of spinal GABA_B_ expression which could be prevented by inhibition of microglia activation [10]. In our current study, we found that chronic oxycodone treatment decreased the activation of glial cells and it was consistent with this report. Co-administration of DM with oxycodone (1 mg/kg, s.c.) reversed the morphology of activated glial cells in SNL mice and further decreased the activated astrocytes and microglia to almost the same level as in the control group (sham + saline) (Figs. 5 and 6). This effect of DM may account for its effect on enhancing the anti-allodynic effect of oxycodone acutely and chronically. Several studies have reported that nerve injury increases expression and secretion of proinflammatory cytokines, TNF-α [49], IL-1β [50], and IL-6 [51], which are required for the development of pain hypersensitivity. It has been also demonstrated that accumulation of morphologically activated glial cells (microglia and astrocytes) releases pro-inflammatory cytokines (TNF-α and IL-1β) in the spinal dorsal horn. TNF-α and IL-1β not only enhance excitatory neurotransmitter (i.e., glutamate) but also suppress inhibitory neurotransmitter release from synaptic terminals and increase the activity of NMDA receptors and its currents in lamina II neurons [52]. It has been reported that DM (12.5 mg/kg, i.p.) inhibits inflammatory mediators, including cytokines (TNF-α, IL-1β and IL-6) and chemokines in sepsis mice model [53]. Furthermore, our recent clinical study showed that DM (60–120 mg/day, oral) attenuated plasma cytokine TNF-α, which has been increased in methadone-treated heroin addicts [54]. Our present study demonstrates that SNL induced high plasma levels of IL-6, IL-1β, and TNF-α, and chronic oxycodone (1 mg/kg, s.c.) suppressed all of them significantly (Fig. 7). DM by itself significantly suppressed the plasma levels of TNF-α and IL-1β but not of IL-6. We also found a trend in which DM potentiated the effect of oxycodone (1 mg/kg, s.c.) on suppressing the plasma levels of IL-6, IL-1β and TNF-α, although this did not achieve statistical significance.

These results suggest that the mechanism of DM for potentiating the chronic effects of oxycodone on suppressing SNL-induced allodynia may be related to its effects on glial and proinflammatory cytokines. On the other hand, we know that the mechanisms for neuropathic pain may involve many factors other than activation of glial cells and inflammatory mediators, such as central sensitization and long-term potentiation (LTP) of nociceptive C-fibers, lamina I neurons and low threshold mechano-sensitive afferents [55, 56], etc. Therefore it is reasonable to see a difference between the degree of the reduction in GFAP-ir or pro-inflammatory cytokines and allodynia as shown in our results.

As we mentioned previously, DM binds to NMDA receptors as a low-affinity non-competitive antagonist and binds to sigma-1 receptor as a high-affinity agonist. NMDA receptors are expressed on both microglia and astrocytes [57], whereas sigma receptors are expressed in microglia [58]. It has been reported that the activation of sigma receptors reduced reactive gliosis following stroke injury in rats [59] and that sigma-1 ligands can modulate several neurotransmitter systems through NMDA receptors [60]. Thus the observed DM effects could be due to both its antagonistic activity on NMDA receptors and agonistic activity on the sigma receptors present in the glial cells.

## Conclusions

In conclusion, our study indicates that DM can potentiate the effects of oxycodone in suppressing SNL-induced allodynia acutely or chronically in mice. Suppression of the overactivation of glial cells (astrocytes and microglia) and the following releases of pro-inflammatory cytokines (IL-6, IL-1β, and TNF-α) might play important roles on this effect. The combination of DM with oxycodone may lower the dose of oxycodone and decrease the side effects of oxycodone, therefore providing beneficial effects in early treatment of neuropathic pain.

## Abbreviations

AAALAC: Association for Assessment and Accreditation of Laboratory Animal Care International
ARRIVE: Animals in Research Reporting *In Vivo* Experiments
DM: Dextromethorphan
SNL: Spinal nerve ligation
NMDA: N-methyl-D-aspartate
AUC: Area under curve
GFAP: Glial fibrillary acidic protein
TNF-α: Tumor necrosis factor alpha
IL: Interleukin
